# Development and Evaluation of ^99m^Tc Tricarbonyl Complexes Derived from Flutamide with Affinity for Androgen Receptor

**DOI:** 10.3390/molecules28020820

**Published:** 2023-01-13

**Authors:** María Elena Cardoso, Paula Decuadra, Maia Zeni, Agustín Delfino, Emilia Tejería, Fátima Coppe, Juan Manuel Mesa, Grysette Daher, Javier Giglio, Gonzalo Carrau, Daniela Gamenara, Omar Alonso, Mariella Terán, Ana Rey

**Affiliations:** 1 Radiochemistry Area, Facultad de Química, Universidad de la República, General Flores 2124, Montevideo CP11800, Uruguay; 2 Organic Chemistry Department, Facultad de Química, Universidad de la República, General Flores 2124, Montevideo CP11800, Uruguay; 3 Centro de Medicina Nuclear e Imagenología Molecular-Hospital de Clínicas, Facultad de Medicina, Universidad de la República, Av.Italia s/n, Montevideo CP11400, Uruguay

**Keywords:** ^99m^Tc, prostate cancer, androgen receptor

## Abstract

With the objective to develop a potential ^99m^Tc radiopharmaceutical for imaging the androgen receptor (AR) in prostate cancer, four ligands bearing the same pharmacophore derived from the AR antagonist flutamide were prepared, labeled with ^99m^Tc, and their structures corroborated via comparison with the corresponding stable rhenium analogs. All complexes were obtained with high radiochemical purity. Three of the complexes were highly stable, and, due to their favorable physicochemical properties, were further evaluated using AR-positive and AR-negative cells in culture. All complexes exhibited considerable uptake in AR-positive cells, which could be blocked by an excess of flutamide. The efflux from the cells was moderate. They also showed significantly lower uptakes in AR-negative cells, indicating interactions with the AR receptor. However, the binding affinities were considerably reduced by the coordination to ^99m^Tc, and the complex that exhibited the best biological behavior did not show sufficient specificity towards AR-positive cells.

## 1. Introduction

The androgen receptor (AR), whose main functions are promoting the growth, differentiation, and survival of epithelial cells in the prostate, plays a fundamental role in prostate cancer. Androgens, such as dihydrotestosterone, bind to the AR, generating a complex that acts as a transcription factor, promoting cell proliferation. Around 80–90% of prostate cancers are androgen-dependent in their early stages, and for this reason, the main therapy consists of androgen receptor inhibition. However, in highly aggressive forms of prostate cancer, there is evidence of a loss of AR expression, and, therefore, these cancers become refractory to androgen suppression therapy. For this reason, it is crucial to determine the presence and distribution of receptors in each patient before and during treatment in order to achieve greater therapeutic efficacy [1]. The development of radiotracers directed at this target has mainly been based on the structures of its steroid agonists, in particular, 16-β-fluoro-5-α-dihydrotestosterone (18F-FDHT). This radiopharmaceutical has shown rapid uptake and prolonged retention in tumors. However, steroid-agonist-based radiopharmaceuticals generally have inadequate selectivity because they have the ability to bind to other steroid hormone receptors [2]. High urinary excretion is also an important drawback since it can impair the detection of lesions.

Another approach for the designing of potential radiopharmaceuticals directed at the AR is the use of non-steroidal antagonists of the receptor, such as flutamide, nilutamide, enzalutamide, and bicalutamide, as leading structures. These antiandrogens possess excellent specificity, selectivity, and pharmacokinetic properties, making them excellent pharmacophores for the development of radiotracers for androgen receptor imaging [3].

There are several examples in the literature of nonsteroidal antiandrogens radiolabeled with positron-emitting radionuclides such as [^18^F]F, [^11^C]C, or [^76^Br]Br. However, the development of technetium-labeled analogs is of paramount importance due to the favorable properties and availability of this radionuclide [4,5,6,7,8]. A limited number of ^99m^Tc-labeled flutamide derivatives have also been prepared and evaluated by various research groups. He et al. [9] prepared a series of potential single-photon emission computed tomography (SPECT) imaging agents on the basis of the structure of flutamide. They conjugated ligands such as histidine, cysteine, and imidazole and prepared the corresponding Tc(I) tricarbonyl complexes. In vitro assays showed limited uptake in AR-positive DU-145 prostate cancer cells. However, further investigations are required to conclusively determine the potential androgen inhibition. Moore et al. [10] compared two approaches using “click” chemistry to prepare flutamide derivatives. This approach allowed the fast, efficient room temperature labeling of M(CO)_3_ (M = Re. ^99m^Tc), which is particularly relevant to temperature-sensitive biomolecules, but no biological evaluation in AR-positive cells or tumor-bearing animals is presented. Dallagi et al. [11] presented a novel cyclopentadienyltricarbonyltechnetium carboxamide flutamide derivative. Although the complex showed a significant selective uptake in the prostate, this was not blocked by an excess of testosterone acetate.

Even if these early attempts to prepare ^99m^Tc-labeled derivatives were not successful, further research should be undertaken since the influences of the chelator and spacer in the biological properties of potential radiopharmaceuticals are well documented in the literature for a variety of molecular targets [12,13,14,15,16].

Our group has a wide experience in the designing of potential ^99m^Tc radiopharmaceuticals bearing the [Tc(CO)_3_]^+^ core for different oncological applications [17,18,19,20]. This core is a very convenient synthon for the labeling of small biomolecules due to its high stability, the diversity of potential donor groups (amines, thioethers, thiols, N-heterocycles, etc.), and the possibility of obtaining radiolabeled molecules with different charges, lipophilicities, and pharmacokinetics [21,22,23,24,25,26].

In this study, we present the syntheses of four flutamide derivatives bearing different chelating units for coordinating ^99m^Tc through the formation of tricarbonyl complexes, as well as the labeling, the structural confirmation using the corresponding stable rhenium complexes, and the evaluation of the main physicochemical and in vitro biological properties.

## 2. Results and Discussion

### 2.1. Syntheses of Flutamide Derivatives

**Figure 1:**
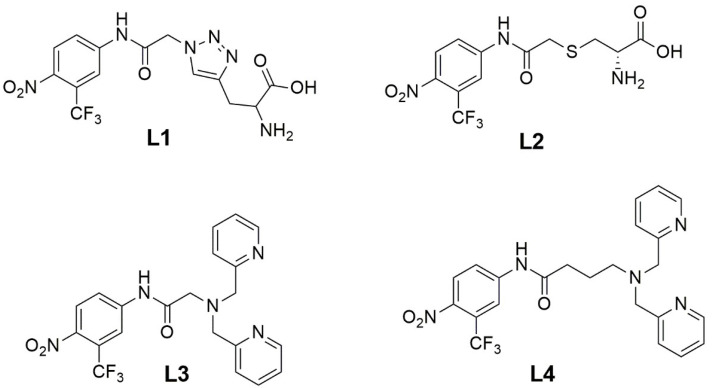
Structures of flutamide derivatives used as ligands for the preparation of ^99m^Tc complexes.

Flutamide (2-methyl-*N*-[4-nitro-3-(trifluoromethyl)phenyl]propanamide) is a nonsteroidal antiandrogen member of the family of trifluoromethyl benzenes that acts as an antagonist of the AR by blocking intracellular androgen receptors in target tissues including those of the testes, prostate, skin, and hair follicles [27,28]. Four derivatives of flutamide (Figure 1) were developed, keeping the essential groups that bind to the receptor, namely, the benzene ring containing the electron-withdrawing substituents, which are the nitro and trifluoromethyl (NO_2_, CF_3_) functional groups.

Different chelating units for Tc bearing aliphatic amines, carboxylate groups, thioethers, and aromatic amines as electron donors were incorporated into the amide group. All of them are tridentate and potentially capable of coordinating Tc in oxidation state I through the formations of tricarbonyl complexes [29,30,31].

**Scheme 1:**
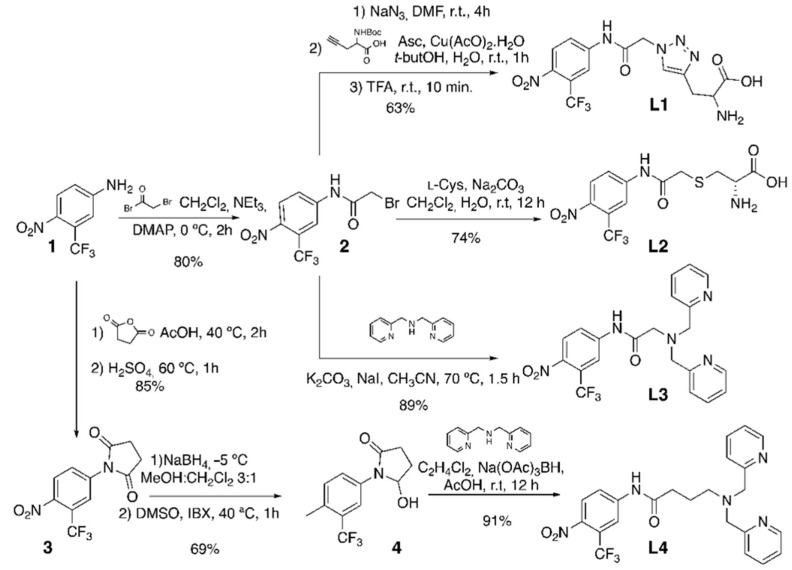
Synthetic schemes for ligands **L1**–**L4**.

**L1**–**L4** were obtained through a divergent route from 4-nitro-3-(trifluoromethyl)aniline (**1**) (Scheme 1). 4-nitro-3-(trifluoromethyl)aniline (**1**) reacted with bromoacetyl bromide according to the literature [32,33] and then the reaction was optimized into 5.3 equivalents of bromoacetyl bromide and 2.4 equivalents of triethylamine per equivalents of **1**. The reaction was carried out at 0 °C, and **2** was obtained with 80% yield. The next step was the substitution of the bromine in **2** for an azide group, and then, a click chemistry reaction was conducted with the Boc-protected propargylglycine, using sodium ascorbate to reduce Cu^+2^ to Cu^+1^ [34]. The last step consisted of the deprotection of the amino group, which was carried out with TFA at room temperature, rendering **L1** as a white solid with 50% global yield from **1**.

Ligand **L2** was prepared by coupling L-cysteine to **2** in a CH_2_Cl_2_:H_2_O biphasic reaction with Na_2_CO_3_ under vigorous stirring for 12 h. **L2** was obtained with 60% global yield from **1** [26,28].

Ligand **L3** was synthesized through a nucleophilic substitution of bis(pyridine-2-ylmethyl)amine on bromide 2 [33]. The reaction was carried out at room temperature with one equivalent of amine per equivalents of 2, K_2_CO_3_, and NaI in MeCN for 1.5 h, rendering **L3** with 89% yield.

The first step in the synthetic sequence to **L4** was the preparation of pyrrolidine 3 by reacting 1 with succinic anhydride in acetic acid at 40° [9,33]. After 2 h, H_2_SO_4_cc was added, and the temperature was raised to 60 °C, obtaining 3 with 85% yield. Then, compound **3** was reduced as previously described. The reaction was carried out with 2 equivalents of NaBH_4_ per equivalent of 3, at −5 °C in MeOH:CH_2_Cl_2_ 3:1. Subsequently, the product was oxidized with 2-iodoxibenzoic acid (IBX) in DMSO at 40 °C, and after 1 h, 4 was obtained with 69% yield. Finally, a reductive amination reaction of 4 was conducted with bis(pyridine-2-ylmethyl)amine as the nucleophile and Na(OAc)_3_BH as the reducing agent. The reaction was carried out at room temperature, with acetic acid in dichloroethane as a reaction solvent. **L4** was obtained with 91% yield (53% global yield from 1).

### 2.2. Preparation of ^99m^Tc Complexes

The labeling of flutamide derivatives was performed through the preparation of ^99m^Tc(I) tricarbonyl complexes, which was achieved via the substitution of **L1** for **L4** on the precursor fac-[ ^99m^Tc(OH_2_)_3_(CO)_3_]^+^. This precursor is ideal for the preparation of ^99m^Tc complexes because of its stability to oxidation and the ease of substitution of the labile coordination positions occupied by the three water molecules with the appropriate donor atom sets of the ligands. Furthermore, it can be prepared at atmospheric pressure and in an aqueous medium using the method developed by Alberto et al. [22].

The substitution was achieved via the incubation of the neutralized precursor with the corresponding ligands dissolved in an appropriate solvent according to their solubility. The radiochemical purities of precursor fac-[^99m^Tc(CO)_3_(OH_2_)_3_]^+^ and final complexes were evaluated with reverse-phase HPLC (RP-HPLC), as described in the experimental section. The mass of the ligand, the volume of the precursor, and the labeling temperature were optimized for each complex.

**L1** (2–6 mg), dissolved in methanol, was reacted with either 100 or 200 µL of the precursor at the standard temperature of 75 °C. Increasing the mass of the ligand from 2 to 3 mg resulted in an increase in the RCP from 60 to 80–85% using 100 µL. An increase in the volume of the precursor, on the other hand, reduced the RCP by about 20% for both masses of the ligand. Further increases in the mass of the ligand from 3 to 5 and 6 mg negatively affected the RCP (70 and 60%, respectively).

**L2** (2–5 mg) was dissolved in acidified water and reacted with the precursor in the same condition as that of **L1**. In this case, the optimal mass of the ligand was 5 mg. Further increases in the mass did not increase the RCP.

**L3** and **L4** were not soluble in water or methanol due to their lipophilic natures, and acetonitrile was the selected solvent. For both ligands (2–6 mg), incubation at 75 °C yielded the corresponding technetium complexes with a low RCP (20–50%) using either 100 or 200 µL of the precursor, which was probably due to the thermal instability of the ligands. The highest RCP in these conditions was achieved using 4 mg of each ligand and 100 µL of the precursor. Consequently, incubation temperatures of 25, 40, and 65 °C were assayed. In both cases, the RCP decreased at higher incubation temperatures (80, 60, and 50%, respectively, for **L3** and 60, 40, and 30%, respectively, for **L4**),

A summary of the best preparation conditions for each complex is shown in Table 1.

**Figure 2:**
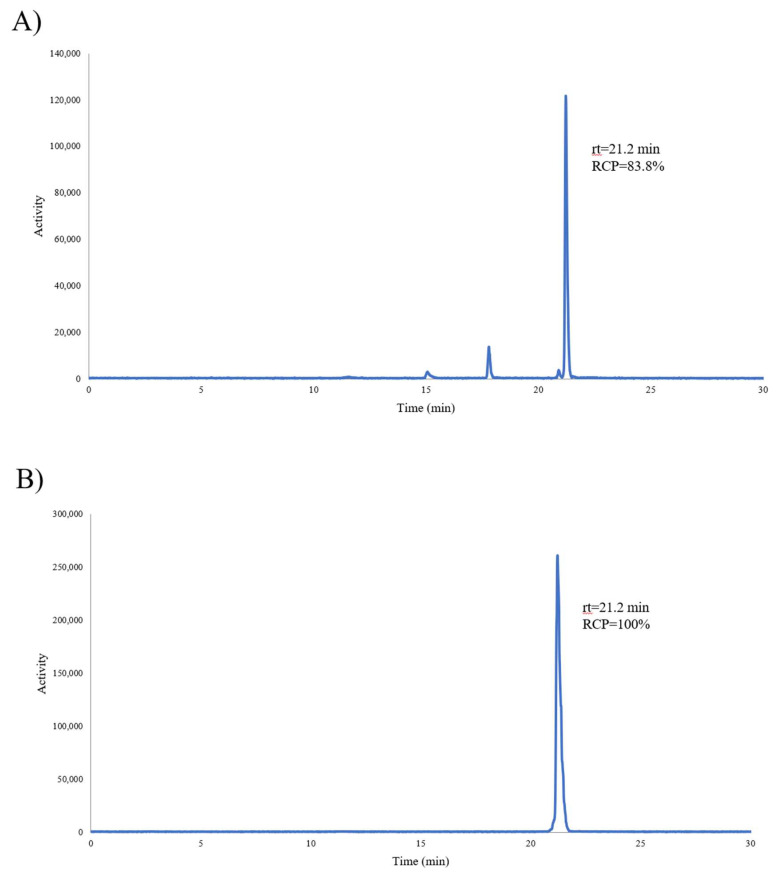
Typical HPLC profile of the radiolabeling corresponding to **C1** (**A**) before and (**B**) after HPLC purification.

HPLC purification was necessary to achieve the required RCP. Figure 2 shows a typical HPLC profile of the radiolabeling before (Figure 2A) and after (Figure 2B) purification, corresponding to **C1**.

### 2.3. Structural Elucidation of the ^99m^Tc Complexes

**Figure 3:**
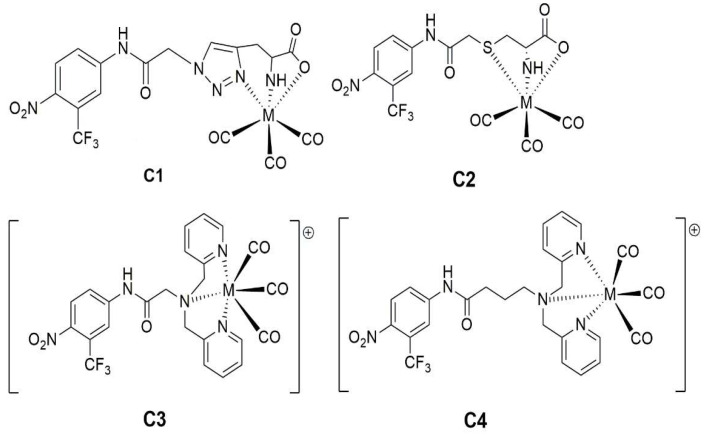
Proposed structures of ^99m^Tc and Re complexes prepared from Ligands **L1**–**L4** (M is either ^99m^Tc or Re).

The structures of the ^99m^Tc complexes were studied using stable rhenium as a surrogate for technetium. Rhenium, as technetium’s third-row congener, exhibits almost identical chemical properties to those of Tc, and, consequently, their respective compounds with the same ligand have essentially equal structures and coordination parameters [35,36]. The analog rhenium complexes were prepared via the substitution of ligands (**L1**–**L4**) on the rhenium precursor fac-[NEt_4_]_2_[Re(CO)_3_Br_3_] [37,38]. Figure 3 shows the proposed structures of the ^99m^Tc/Re complexes.

The HPLC analyses with UV detection of the reaction mixtures showed in all cases the main peaks with the same retention time of the corresponding technetium complexes. The rhenium complexes were isolated using semipreparative HPLC, and the MS spectra of the purified compounds were consistent with the proposed structures and matched the isotopic distribution of stable rhenium. The results are shown in Table 2.

Ligand 1 is tridentate and has a histidine-like donor atom set formed by an imidazolic nitrogen [31], an aliphatic amine nitrogen, and a carboxylate oxygen from a carboxylic acid. 1,4-disubstituted 1,2,3-triazoles, synthesized with the so-called click chemistry starting from propargylglycine, share structural and electronic features with 1,4-disubstituted imidazoles of *N*^ε^-derivatized histidines, which have been shown to be extraordinarily good chelators, particularly for the organometallic cores of Mo, Tc, and Re.

Ligand 2 is a cysteine derivative that bears an NSO-type chelator unit, similar to the homocysteine derivative developed by Karagiorgou et al. [38], in which the donor-atom system consists of a *N*-primary amine, a *S*-thioether, and an *O*-carboxylate. It is well known that *S*-alkylated cysteine derivatives bind efficiently with the tricarbonyl core via its NSO donor atom set to provide stable and neutral complexes. Coordination affords two five-membered and one six-membered ring providing thermodynamically stable hexacoordinated complexes [39].

Ligands 3 and 4 contain essentially the same tridentate N3 chelator formed by two pyridines and one tertiary amine, resulting in positively charged compounds. This chelator binds avidly to the {M(CO)_3_}^+^ core (M = ^99m^Tc or Re) as reported in the literature and has been used for the preparation of different biomolecules such as peptides, carbohydrates, etc. The difference between both ligands is in the length of the aliphatic chain spacer (either 1 or 3 methylene groups), and we intended to study the influence of the length of the spacer on the chemical and biological properties of the final technetium complexes.

### 2.4. Physicochemical Evaluation

The physicochemical evaluation included stability studies in different conditions, lipophilicity, and plasma protein binding.

#### 2.4.1. Stability

Stability in the labeling milieu, in human plasma, and after incubation with histidine as a competitive ligand were assessed with HPLC. All complexes were stable in the labeling milieu for up to 4 h. Complexes **C1**, **C3**, and **C4** were also stable after incubation in human plasma for at least 4 h with an RCP higher than 90%. Stability against ligand exchange was studied via incubation with a 100-fold molar excess (7.3 × 10^−3^ M) of histidine for 2 h. No signs of transchelation or instability were observed for these three complexes. The results are shown in Table 3.

However, **C2** was less stable than the other complexes. The RCP remained higher than 90% up to 2 h of incubation in human plasma but decreased to 75% after 3 h. **C2** also showed remarkable instability against transchelation. RCP decreased from almost 100% to 73% after 1 h of incubation and to less than 30% after 4 h. This result was unexpected since this donor atom set has been successfully applied by our group to the design of a potential hypoxia-imaging agent [33]. Furthermore, other authors [9,39] also describe the same chelator with no mention of any instability.

#### 2.4.2. Protein Binding

Plasmatic protein binding (PPB) was assessed using size-exclusion chromatography at 0.5 and 1 h. Moderate PPB is a positive property for potential radiopharmaceuticals since only the unbound fraction of the complex can penetrate the cells. Table 3 shows the results obtained for **C1**, **C3**, and **C4**. The three complexes exhibited low–moderate values (14–42%) and no significant difference between the two time points studied. When compared with flutamide, which has a high PPB (95 ± 1%), all the complexes presented clearly lower values. These results show the impact of the donor atoms set on the overall physicochemical behavior of Tc-labeled small biomolecules, a fact that has been corroborated by our group and other research groups before [14,15,17]. A comparison of **C3**, which has the lowest PPB, with **C4**, which has the highest value, also demonstrates that the length of the linker has, as expected, a considerable impact on the physicochemical properties of the labeled compounds.

Once again, **C2** exhibited a completely different behavior (Table 4), and its PPB was low at the 30 min incubation time but increased remarkably with time, which was probably due to the substitution of one or all donor atoms from the ligand for electron donor groups from the proteins that were present at a considerably higher concentration in relation to the technetium complex. This result is consistent with the instability towards transchelation with histidine described in the previous section.

#### 2.4.3. Lipophilicity

Lipophilicity was assessed through the determination of the partition coefficient between 1-octanol and a phosphate buffer 0.1 M (pH 7.4), as shown in Table 3. All complexes were moderately lipophilic with values in the adequate range to cross the cell membrane, considering that the optimal values of Log P for diffusion across biological membranes are between −0.5 and 2.0 [40]. This feature is very important since the androgen receptor is located inside the cell, and it is necessary for the complex to cross the phospholipid membrane to interact with it [41]. When compared with flutamide (Log P = 3.35), all complexes are less lipophilic, probably due to the addition of hydrophilic groups for coordination and, in the cases of **C3** and **C4**, due to their overall positive charge.

### 2.5. In Vitro Biological Evaluation

The in vitro biological behaviors of **C1**, **C3**, and **C4** were assessed via uptake, efflux, and binding affinity studies using cells in culture. Studies with **C2** were not performed due to the instability detected during the physicochemical evaluation.

#### 2.5.1. Uptake Studies

The uptakes of the ^99m^Tc complexes were evaluated in LNCaP cells at 1, 2, and 4 h of incubation. The results are shown in Table 5. LNCaP cells are derived from metastatic human prostatic adenocarcinoma and were selected because they are the standard AR-positive cells. All the cellular uptake values were remarkably high, which could be attributed to their lipophilic characters. According to the literature lipophilic radiotracers tend to bind easily not only to their molecular targets but also non-specifically to other molecules [40]. The uptake values did not show significant variations over time during the studied period.

Additional uptake studies were performed to establish the potential implication of the androgen receptor in the previous results. The inhibition of the uptakes of the radiotracers via competition with flutamide, the AR antagonist used as the lead structure to design the ^99m^Tc complexes of this study, was determined. Additionally, uptake in PC3 cells, androgen-independent prostate carcinoma cells, was also assessed as a negative control. The comparative results of the three uptake studies displayed in Table 6 show that the uptakes of the three ^99m^Tc complexes were significantly reduced via the competition with flutamide, resulting in a decrease in uptakes from 50% to 70% of the initial values. This result indicates that a specific interaction could be responsible for the uptakes of these complexes by the AR-positive cells. On the other hand, uptake in PC3 cells was in all cases significantly lower than uptake in LNCaP cells, reinforcing our hypothesis of a specific AR-mediated uptake. Furthermore, the uptake in PC3 cells was in the same range as the nonspecific uptake in LNCaP cells for **C1** and **C4**. Surprisingly, **C3** exhibited a very high uptake in PC3 cells, significantly higher than the uptake in blocked LNCaP cells (nonspecific binding). **C3** also showed a very high uptake in unblocked LNCaP cells. The high uptake in both cell lines does not correlate with the lipophilicity, which was very similar to the other complexes. **C3** also had the lowest protein binding and good stability. In conclusion, this complex seems to have a significant uptake by the androgen receptor but also has another unknown AR-independent mechanism of uptake in PC3 cells.

#### 2.5.2. Efflux Studies

Efflux studies were performed to measure the release of the radiotracers from the cells. A rapid and high efflux indicates low target retention, while a low release could be an indicator that the complex could be retained for long periods of time in the target tissue, and the results are shown in Table 7. The rate of released radiotracer from cells was moderated for the 3 complexes and remained constant during the period under study with values between 20 to 50% of the total initial activity taken up by cells. **C3** exhibited the lowest efflux with more than 80% of the initial activity taken up by AR-positive cells retained after 4 h.

#### 2.5.3. Binding Affinity Studies

**Figure 4:**
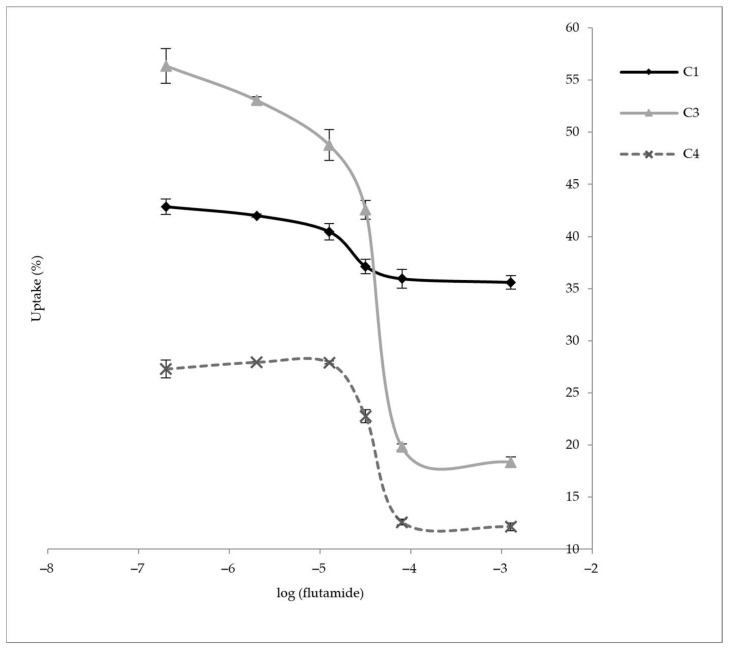
Binding assay graphs for complexes **C1** (grey), **C3** (black), and **C4** (dotted line).

The binding affinities of the radiolabeled flutamide derivatives were assessed in a competition assay against increasing concentrations of flutamide. The IC_50_ values were calculated using a non-linear regression, as shown in Figure 4.

All complexes exhibited IC_50_ values in the micromolar range and of approximately two orders of magnitude higher than flutamide (IC_50_ 0.9 µM), and these results are summarized in Table 8. The derivatization of flutamide to introduce the different donor groups resulted in reductions in the affinities, which in this case were slightly influenced by the natures of the donor atom sets.

In summary, **C3** had the highest specific uptake, the lowest efflux percentage, and the highest binding affinity. This can be explained by taking into consideration that according to the literature [9], the electron-withdrawing groups of the aromatic ring, namely NO and CF3, and the amide group stabilize and promote the binding to the AR. Particularly, the oxo group of the amide has a key role, as it forms hydrogen bonds with *Leu873* of the AR through a water molecule precluded in the structure [42,43]. The modifications introduced during the derivatization generate more bent ligand conformations than flutamide as the interaction of the metal cores with the AR backbone is altered. **C1** and **C4** may present a steric hindrance to preclude the water molecule and thus have fewer hydrogen bonds with the receptor, while the chiral center of **C3** allows these kinds of interactions favoring the retention of the complex inside the cells.

## 3. Materials and Methods

### 3.1. General

All non-hydrolytic reactions were carried out in a nitrogen atmosphere using standard techniques for the exclusion of air. All solvents were distilled prior to use. Starting materials and reagents were purchased from commercial suppliers and were used without further purification, unless otherwise stated. NMR spectra were acquired using a Bruker Ascend 400 MHz or a Bruker Avance DPX 400 MHz (Berlin, Germany). All experiments were undertaken at 30 °C and using solvents indicated in each case. Proton chemical shifts (δ) are reported in parts per million (ppm) downfield from TMS as an internal reference, and carbon chemical shifts are reported in ppm relative to the center line of the CDCl_3_ triplet (δ = 77.0 ppm). Analytical TLC was performed on Silica gel 60F-254 plates and visualized with UV light (245 nm) and/or *p*-anisaldehyde or vanillin in an acidic ethanolic solution. Flash column chromatography was performed using silica gel-flash (Macherey Nagel 60, 0.040–0.063 mm, Allentown, PA, USA).

[^99m^Tc] NaTcO_4_ was obtained from a commercial ^99^Mo/^99m^Tc generator (Tecnonuclear, Buenos Aires, Argentina). Solvents for the chromatographic analysis were HPLC-grade. Activity measurements were performed either with a dose calibrator (Capintec CRC-5R, Florham Park, NJ, USA) or with a scintillation counter (3 × 3 in. NaI (Tl) crystal detector) attached to an ORTEC monochannel analyzer (ORTEC, Buenos Aires, Argentina).

The HPLC analysis was developed with a UltiMate3000 Dionex (Conquer Scientific, Poway, CA, USA) coupled to a Berthold HERM gamma detector (Baden-Württemberg, Germany) and an UltiMate 3000 UV.vis detector (Thermo Fisher, Waltham, MA, USA) using a reverse-phase column. Elution was performed with a binary gradient system at a 1.0 mL/min flow rate using a triehtylamine-phosphate buffer (pH 2.5) as mobile phase A and methanol as mobile phase B; the elution profile was as follows: 0–3 min 100% A; 3–6 min linear gradient to 25% B; 6–9 min linear gradient to 34% B; 9–20 min linear gradient to 100% B; 20–27 min 100% B; and 27–30 min linear gradient 0% B. Mass spectra were recorded using a mass spectrometer API 2000 LC/MS/MS System (SpectraLab Scientific, St. Markham, ON, Canada).

### 3.2. Syntheses of Flutamide Derivatives

**2-Bromo-*N*-[4-nitro-3-(trifluoromethyl)phenyl]acetamide (2)**. A solution of **1** (0.2 g, 0.97 mmol) in CH_2_Cl_2_ (6 mL) and triethylamine (0.74 mL, 5.15 mmol) was cooled to 0 °C in a two-neck round-bottom flask under an N_2_ atmosphere. Bromoacetyl bromide (0.48 g, 2.38 mmol) was added dropwise, and, finally, a catalytic amount of DMAP was added. The solution was stirred for 2 h and washed with HCl 0.5 M (3 × 10 mL) and a saturated aqueous NaCl solution (2 × 10 mL). The organic phase was dried over anhydrous Na_2_SO_4_, filtered, and the solvent was distilled under reduced pressure. The residue was purified using flash column chromatography (CH_2_Cl_2_:Hexane 8:2), yielding **2** as a colorless oil (0.277 g, 80%).

^1^H RMN (400 MHz, CDCl_3_) δ (ppm): 8.50 (s, 1H), 8.02 (m, 3H), and 4.07 (s, 2H). ^13^C RMN (101 MHz, CDCl_3_) δ (ppm): 164.21, 141.20, 127.22, 125.55 (q, *J* = 34.4 Hz), 122.59, 121.74 (q, *J* = 273.6 Hz), 118.61 (q, *J* = 5.8 Hz), and 29.03.

**2-amino-3-(1-(2-((4-nitro-3-(trifluoromethyl)phenyl)amino)-2-oxoethyl)-1*H*-1,2,3-triazol-4-yl)propanoic acid (L1).**

S_N_2 azide substitution: To a solution of **2** (0.295 g, 0.903 mmol) in DMF (1 mL), sodium azide (0.352 mL, 5.418 mmol) was added, and the reaction was stirred at room temperature for 4 h in a two-neck round-bottom flask under a N_2_ atmosphere. The crude was diluted with H_2_O (10 mL), extracted with Et_2_O (3 × 10 mL), washed with an aqueous NaCl solution (2 × 10 mL), and dried over anhydrous Na_2_SO_4_. The solvent was distilled under reduced pressure, and the residue was purified using flash column chromatography (Hexane:AcOEt 7:3) (0.246 g, 94%). ^1^H RMN (400 MHz, CDCl_3_) δ (ppm): 8.50 (s, 1H), 8.02 (m, 3H), 4.24 (s, 2H), and 1.25 (s, 1H). ^13^C RMN (101 MHz, CDCl_3_) δ (ppm): 165.37, 141.08, 127.25, 125.48 (q, *J* = 34.2 Hz), 122.60, 121.74 (q, *J* = 274.7 Hz), 118.64 (q, *J* = 5.8 Hz), and 52.9.

Cu-catalyzed alkyne-azide cycloaddition: To a solution of the azide-containing compound (0.672 g, 2.32 mmol) in 24 mL of *t*-butanol, 2-((*t*-butoxycarbonyl)amino)pent-4-ynoic acid (0.495 g, 2.32 mmol) was added. Separately, Cu(OAc)_2_·H_2_O (0.093 g, 0.46 mmol) was added to a solution of sodium ascorbate (0.176 g, 0.93 mmol) in H_2_O (12 mL), and this solution was immediately poured into the reaction flask. The reaction was stirred for 1 h at room temperature until the starting material was consumed (as determined using TLC). The solvent was distilled under reduced pressure, and the residue was taken up in AcOEt (20 mL). The organic layer was washed with HCl 0.5 M (3 × 20 mL), dried over anhydrous Na_2_SO_4_, filtered, and the solvent was distilled under reduced pressure. The residue was purified using flash column chromatography (CH_2_Cl_2_:MeOH 98:2) (0.691 g, 70%). ^1^H NMR (400 MHz, Metanol-*d*_4_) δ (ppm): 8.24 (d, *J* = 2.2 Hz, 1H), 8.05 (d, *J* = 8.9 Hz, 1H), 7.99 (dd, *J* = 8.9, 2.3 Hz, 1H), 7.86 (s, 1H), 5.37 (s, 1H), 4.34 (s, 1H), 3.29–3.09 (m, 1H), and 1.41 (s, 9H). ^13^C NMR (101 MHz, Metanol-*d*_4_) δ (ppm): 166.80, 143.97, 128.20, 126.22, 125.66 (q, *J* = 33.7 Hz), 124.81 (q, *J* = 271.6 Hz), 123.85, 119.16 (q, *J* = 5.9 Hz), 80.46, 53.57, 29.55, and 28.70. Boc deprotection: TFA (0.5 mL) was added to the previously obtained triazol compound (0.025 g, 0.0622 mmol) at room temperature, and the reaction was stirred for 10 min. The TFA was distilled under reduced pressure, then a mixture of CH_2_Cl_2_:MeOH (9:1) (containing 1% of concentrated NH_4_OH) was added, and redistilled under reduced pressure. This procedure was repeated until **L1** was obtained as a white solid (0.018g, 96%). ^1^H NMR (400 MHz, D_2_O) δ (ppm): 8.09–7.87 (m, 2H), 7.87–7.73 (m, 1H), 5.53–5.46 (m, 2H), 4.37–4.29 (m, 1H), and 3.52–3.38 (m, 2H). ^13^C NMR (101 MHz, D_2_O) δ (ppm): 171.83, 166.65, 141.60, 127.37, 126.28, 114.86, 53.33, 52.43, and 25.93.

**(2-(*S*-cysteinyl)-*N*-[4-nitro-3-(trifluoromethyl)-phenyl]-acetamide) (L2):** To a solution of **2** (0.157 g, 0.482 mmol) in CH_2_Cl_2_ (8 mL), a solution of L-Cysteine (0.117 g, 0.964 mmol) in H_2_O (8 mL) and NaHCO_3_ (0.045 g, 0.530 mmol) were added. The biphasic mixture was stirred vigorously for 12 hs at room temperature. The solvent was distilled under reduced pressure, and the residue was purified using flash column chromatography (CH_2_Cl_2_:MeOH 9:1), yielding **L2** (0.130 g, 74%). ^1^H RMN (400 MHz, DCl 0.25 M in D_2_O) δ (ppm): 7.97 (d, *J* = 9.0 Hz, 1H), 7.95 (d, *J* = 2.3 Hz, 1H), 7.79 (dd, *J* = 2.3, 8.96 Hz, 1H), 4.28 (dd, *J* = 7.9, 4.3 Hz, 1H), 3.54 (d, *J* = 15.8 Hz, 1H), 3.49 (d, *J* = 15.8 Hz, 1H), 3.27 (dd, *J* = 15.01, 4.4 Hz, 1H), and 3.12 (dd, *J* = 15.1, 7.9 Hz, 1H). ^13^C NMR (101 MHz, DCl 0.25 M in D_2_O) δ (ppm): 170.47, 170.13, 142.18, 142.05, 127.27, 123.96 (q, *J* = 33.7 Hz), 123.04, 121.64 (q, *J* = 273.2 Hz), 118.77 (q, *J* = 5.8 Hz), 52.11, 36.13, and 31.96.

**2-(*bis*(pyridin-2-ylmethyl)amino)-*N*-(4-nitro-3-(trifluoromethyl)phenyl)acetamide (L3):** A solution of bis(pyridin-2-ylmethyl)amine (0.040 g, 0.20 mmol), **2** (0.065 g, 0.20 mmol), K_2_CO_3_ (0.055 g, 0.40 mmol), and NaI (0.06 g, 0.40 mmol) in dried CH_3_CN was stirred at 70 °C for 1.5 h. The solvent was distilled under reduced pressure, and the crude was dissolved in CH_2_Cl_2_, and the solution was washed with an aqueous NaCl solution (3 × 10 mL) and dried over anhydrous Na_2_SO_4_. The residue was purified using column chromatography (CH_2_Cl_2_:MeOH 95:5/NH_4_OH (1%)), yielding **L3** (0.085 g, 89%). ^1^H NMR (400 MHz, MeOD) δ (ppm): 11.83 (s, 1H), 8.63 (dt, *J* = 4.7, 1.5 Hz, 2H), 8.39 (dd, *J* = 8.9, 2.3 Hz, 1H), 8.22 (d, *J* = 2.3 Hz, 1H), 8.01 (d, *J* = 8.9 Hz, 1H), 7.65 (dt, *J* = 7.7, 1.8 Hz, 2H), 7.31 (m, 4H), 3.94 (s, 4H), and 3.58 (s, 2H). ^13^C NMR (101 MHz, CDCl_3_) δ (ppm): 171.25, 157.84, 149.55, 143.37, 142.56, 137.01, 127.37, 125.16 (q, *J* = 33.8 Hz), 123.54, 123.02, 122.16 (q, *J* = 273.4 Hz), 122.04, 118.53 (q, *J* = 5.9 Hz), 60.20, and 58.89.

**1-(4-nitro-3-(trifluoromethyl)phenyl)pyrrolidine-2,5-dione (3)**. Succinic anhydride (0.515 g, 4.85 mmol) was added to a solution of **1** (0.5 g, 2.45 mmol) in AcOH (2 mL). The reaction was stirred for 2 h at 40 °C, then H_2_SO_4_ cc (260 mL) was added, the temperature was raised to 60 °C, and the reaction was stirred for an additional hour. The reaction was diluted with H_2_O (5 mL), the organic layer was extracted with AcOEt (3 × 5 mL), washed with saturated aqueous NaCl solution (2 × 5 mL), dried over anhydrous Na_2_SO_4_, filtered, and the solvent was distilled under reduced pressure. The residue was purified using flash column chromatography (Hexane:AcOEt 6:4), yielding **3** (0.597 g, 85%). ^1^H NMR (400 MHz, Acetone-*d*_6_) δ (ppm): 8.26 (d, *J* = 8.6 Hz, 1H), 8.04 (d, *J* = 2.2 Hz, 1H), 7.99 (dd, *J* = 8.7, 2.1 Hz, 1H), and 2.95 (s, 4H). ^13^C NMR (101 MHz, Acetone-*d*_6_) δ (ppm): 176.68, 137.97, 132.51, 127.13, 126.72 (q, *J* = 5.6 Hz), 123.93 (q, *J* = 34.2 Hz), 122.93 (q, *J* = 272.7 Hz), and 29.29 (s, 4C).

**5-hydroxy-1-(4-nitro-3-(trifluoromethyl)phenyl)pyrrolidin-2-one (4)**. NaBH_4_ reduction: To a solution of **3** (0.522 g, 1.80 mmol) in a MeOH:CH_2_Cl_2_ 3:1 mixture (40 mL), NaBH_4_ (0.135 g, 3.2 mmol) was added in three portions at −5 °C. The reaction was diluted with H_2_O (20 mL), the MeOH was distilled under reduced pressure, and the aqueous phase was extracted with AcOEt (3 × 20 mL). The organic layer was washed with a saturated aqueous NaCl solution (2 × 5 mL), dried over anhydrous Na_2_SO_4_, filtered, and the solvent was distilled under reduced pressure. The residue was purified using flash column chromatography (Hexane:AcOEt 4:6) (0.400 g, 76%). ^1^H NMR (400 MHz, Acetone-*d*_6_) δ (ppm): 9.91 (s, 1H), 8.35 (s, 1H), 8.12 (s, 2H), 3.75–3.67 (m, 1H), 3.62 (q, *J* = 5.7 Hz, 2H), 2.57 (t, *J* = 7.4 Hz, 2H), and 1.95–1.83 (m, 2H). ^13^C NMR (101 MHz, Acetone-*d*_6_) δ (ppm): 173.44, 144.99, 142.99, 128.26, 124.86 (q, *J* = 33.7 Hz), 123.33 (d, *J* = 272.5 Hz), 122.91, 118.31 (q, *J* = 6.1 Hz), 61.70, 34.58, and 29.00.

IBX oxidation: IBX (0.910 g, 3.26 mmol) was added to a solution of the obtained product (0.636 g, 2.18 mmol) in DMSO (10 mL), and the mixture was stirred for 1 h at 40 °C. The reaction was diluted with H_2_O (10 mL) and extracted with AcOEt (3 × 20 mL). The organic layer was washed with a saturated aqueous NaCl solution (2 × 5 mL), dried over anhydrous Na_2_SO_4_, and the solvent was distilled under reduced pressure. The crude was purified using flash column chromatography (Hexane:AcOEt 6:4 to 4:6), yielding **4** (0.577 g, 91%). ^1^H NMR (400 MHz, Acetone-*d*_6_) δ (ppm): 8.51 (d, *J* = 2.3 Hz, 1H), 8.24 (dd, *J* = 9.0, 2.4 Hz, 1H), 8.16 (d, *J* = 9.0 Hz, 1H), 5.98 (d, *J* = 5.7 Hz, 1H), 5.87 (s, 1H), 3.07–2.73 (m, 2H), 2.54 (d, *J* = 1.8 Hz, 1H), and 2.16–2.06 (m, 1H). ^13^C NMR (101 MHz, Acetone-*d*_6_) δ (ppm): 175.32, 143.87, 127.45, 124.91, 124.18 (q, *J* = 33.5 Hz), 123.23 (d, *J* = 272.5 Hz), 119.94 (q, *J* = 5.9 Hz), 84.86, 30.61, and 28.78.

**4-(bis(pyridin-2-ylmethyl)amino)-*N*-(4-nitro-3-(trifluoromethyl)phenyl)butanamide (L4)**. To a solution of **4** (0.577 g, 1.99 mmol), Na(OAc)_3_BH (1.51 g, 7.16 mmol), and AcOH (237 µL, 4.17 mmol) in dichloroethane (10 mL), bis(pyridin-2-ylmethyl)amine (0.516 g, 2.58 mmol) was added. The reaction was stirred for 12 h at room temperature. The crude was diluted with H_2_O (10 mL), and the mixture was extracted with AcOEt (3 × 10 mL). The organic layer was washed with a saturated aqueous NaCl solution (2 × 5 mL), dried over anhydrous Na_2_SO_4_, and the solvent was distilled under reduced pressure. The crude was purified using flash column chromatography (AcOEt:MeOH 98:2), yielding **L4** (0.850 g, 91%). ^1^H NMR (400 MHz, Acetone-*d*_6_) δ (ppm): 10.40 (s, 1H), 8.48 (ddd, *J* = 4.9, 1.9, 1.0 Hz, 2H), 8.30 (d, *J* = 2.1 Hz, 1H), 8.09 (d, *J* = 9.0 Hz, 1H), 8.05 (dd, *J* = 8.9, 2.2 Hz, 1H), 7.66 (td, *J* = 7.6, 1.9 Hz, 2H), 7.51 (dt, *J* = 7.9, 1.1 Hz, 2H), 7.18 (ddd, *J* = 7.6, 4.9, 1.2 Hz, 2H), 3.80 (s, 4H), 2.61 (t, *J* = 6.6 Hz, 2H), 2.51 (t, *J* = 7.0 Hz, 2H), and 2.01–1.91 (m, 2H). ^13^C NMR (101 MHz, Acetone-*d*_6_) δ (ppm): 173.53, 160.42, 149.72, 145.12, 142.72, 137.16, 128.09, 124.70 (q, *J* = 33.6 Hz), 124.04, 123.24 (q, *J* = 272.5 Hz), 122.86, 122.80, 118.22 (q, *J* = 6.1 Hz), 60.75, 54.11, and 23.52.

### 3.3. Syntheses of ^99m^Tc Complexes

#### 3.3.1. Synthesis of fac-[^99m^Tc(CO)_3_(H_2_O)^3^]^+^

The ^99m^Tc-precursor complex [14], was prepared according to a previously described method as follows: Na/K tartrate (20.0 mg), Na_2_CO_3_ (4.0 mg), and NaBH4 (7.0 mg) were placed in a vial. The vial was sealed and flushed with carbon monoxide for 30 min. ^99m^Tc-sodium pertechnetate (185–1850 MBq, 1 mL) was added, and the mixture was incubated at 75 °C for 30 min. After cooling, the pH was adjusted to 7.0 with 1N HCl solution. The complex formation was monitored with HPLC analysis as indicated in the general experimental section.

#### 3.3.2. Substitution with Ligands **L1**–**L4**

The neutralized fac-[^99m^Tc(OH_2_)_3_(CO)_3_]^+^ precursor (100–200 μL, 185–1850 MBq) was incubated with the corresponding ligand dissolved in the appropriate solvent. The amount of ligand, incubation time, and temperature were optimized to achieve the highest radiochemical purity. The optimal conditions are shown in Table 1. Complex formation was monitored with HPLC, as indicated in the general experimental section. Purification was performed using HPLC under the same conditions.

### 3.4. Structural Elucidation

Stable rhenium analogs of **C1**–**C4** were prepared as follows: a solution of **C1**–**C4** (64 × 10^−6^ moles) in either water or methanol according to the solubility of each ligand was mixed with the precursor fac-[NEt_4_]_2_[Re(CO)_3_Br_3_] in the same solvent and reacted at reflux for 5 h. Progress was monitored using HPLC coupled to a UV detector in the conditions described in the general experimental section. The same chromatographic system was used to purify the complexes. The identity of the compounds was assessed using mass spectrometry. The retention times were compared with the corresponding ^99m^Tc complexes.

### 3.5. Physicochemical Evaluation

#### 3.5.1. Stability after HPLC Purification

The HPLC-purified ^99m^Tc complexes **C1**–**C4** were incubated at room temperature, and radiochemical purity was checked with HPLC analysis as described previously for up to 4 h after labeling.

#### 3.5.2. Stability in Human Plasma

The ^99m^Tc complexes (**C1**–**C4**, 100 µL), in human plasma (900 µL), were incubated at 37 °C for up to 4 h. After different incubation times (1, 2, 3, and 4 h), the samples (200 µL) were precipitated with cold ethanol (200 µL) and incubated at −15 °C for 5 min. After centrifugation at 15,000 rpm for 5 min at 0 °C, the pellets were carefully separated from the supernatant, and an aliquot of the latter was analyzed using HPLC as described previously.

#### 3.5.3. Stability against Histidine

The ^99m^Tc complexes (**C1**–**C4**, 100 µL) were mixed with 900 µL of a solution containing a 100-fold molar excess of histidine and incubated at 37 °C. At 1, 2, 3, and 4 h, 200 µL samples were withdrawn, and the variation in RCP was evaluated using HPLC with the reference system.

#### 3.5.4. Lipophilicity

Lipophilicity was studied through the apparent partition coefficient (P) between n-octanol and the sodium phosphate buffer (0.1 M, pH 7.4). In a centrifuge tube containing 2.0 mL of n-octanol, 1.9 mL of the phosphate buffer, and 0.1 mL of a complex, the mixture was shaken with a Vortex mixer for 2 min and finally centrifuged at 5000 rpm for 5 min. The activity of samples of 100 μL from each phase (n = 3) was measured with a solid scintillation counter. The partition coefficient (P) was calculated as the mean value of each Bq/mL of the n-octanol layer divided by that of the buffer. Lipophilicity was expressed as log P.

#### 3.5.5. Plasma Protein Binding (PPB)

An amount of 25 µL of either ^99m^Tc complexes (**C1**–**C4**) or distilled water as a blank was incubated with fresh human plasma (475 µL) in a water bath at 37 °C. At 30 and 60 min, 25 µL samples were seeded in a size-exclusion chromatography column (Microspin G-50, GE Healthcare, Buckinghamshire, UK). Columns were centrifuged at 4000 rpm for 1 min, and the activity of the eluate and the column was measured with a solid scintillation counter. The protein-bound tracer was calculated as the percentage of activity eluted from the column.

### 3.6. Biological In Vitro Evaluation

To complete the evaluation of complexes **C1**, **C3**, and **C4**, in vitro biological behavior was assessed. Two different cell lines derived from prostatic adenocarcinoma were used, LNCaP, which express the AR, and PC3 cells, which do not express it.

The adherent cell line LNCaP (ATCC^®^ CRL-1740™, American Type Culture Collection, Manassas, VA, USA) was cultured in a conditioned RPMI 1640 medium (Capricorn Scientific, Ebsdorfergrund, Germany) supplemented with 10% fetal bovine serum (Capricorn Scientific), 100 U/mL of penicillin (Sigma-Aldrich, St. Louis, MI, USA), and 100 μg/mL of streptomycin (Sigma-Aldrich) in T75 flasks (Greiner Bio-one, Frickenhausen, Germany) at 37 °C and 5% CO_2_. The studies were performed with monolayer cultures with less than 25 passages.

The adherent cell line PC3 (ATCC^®^ CRL-1435™, American Type Culture Collection, Manassas, VA, USA) was cultured in a DMEM medium (A1316, 9050 PanReac AppliChem, ITW Reagents, Darmstadt, Germany) supplemented with 10% fetal bovine serum, 100 U/mL of penicillin, and 100 μg/mL of streptomycin in T75 flasks (Greiner Bio-one, Kremsmünster, Austria) at 37 °C and 5% CO_2_. The studies were performed with monolayer cultures with less than 20 passages.

Cellular uptake, efflux, and binding studies for each complex were performed.

#### 3.6.1. Cellular Uptake

LNCaP or PC3 (monolayer, equivalent to approximately 7 × 10^6^ cells per flask) was incubated with **C1**, **C2**, and **C3** (0.925 MBq, 25 µCi) for 1, 2, and 4 h at 37 °C and 5% CO_2_ (n = 4). After incubation, the culture medium was removed, and cells were washed twice with PBS (10 mL) and treated with trypsin-EDTA (3 mL, 5 min, 37 °C). For each complex and time point, the activity in the supernatant and bound to the cells were measured with a solid scintillation counter. The cellular uptake was expressed as the percentage of activity in the cells over the total activity (Equation (1)).

(1) $$\%\mathrm{cellular}\;\mathrm{uptake}=\frac{\mathrm{Act}.\;\mathrm{in}\;\mathrm{cells}}{\mathrm{Act}.\mathrm{in}\;\mathrm{cells}+\mathrm{Act}.\;\mathrm{in}\;\mathrm{supernatant}}\times 100$$

LNCaP cells (monolayer, equivalent to 7 × 10^6^ cells per flask) were incubated for 1 h (37 °C, 5% CO_2_) with a fixed activity of each complex under study (0.925 MBq, 25 µCi) and a high concentration of flutamide (1.2 × 10^−3^ M) to determine the nonspecific uptake. After incubation, the culture medium was removed, and cells were washed with PBS and treated with trypsin (5 min, 37 °C). The activity in the supernatant and the activity bound to the cells were measured. % Cellular uptake was calculated using Equation (1).

#### 3.6.2. Efflux Studies

LNCaP cells were incubated with each complex in the same conditions as described previously (monolayer, equivalent to 7 × 10^6^ cells per flask). After incubation, the culture medium was removed, and cells were washed twice with PBS (10 mL), a new culture medium (12 mL) was added, and the cells were re-incubated for 1, 2, and 4 h. At each time point, the culture medium was removed, washed twice with PBS, and treated with trypsin-EDTA. Activity in the supernatant and cell-bound activity were measured. The efflux is expressed as the percentage of activity originally absorbed by cells that diffuses out of the cells and is recovered in the supernatant over the total activity (Equation (2)).

(2) $$\%\mathrm{Efflux}=\frac{\mathrm{Act}.\;\mathrm{supernatant}}{\mathrm{Act}.\;\mathrm{in}\;\mathrm{cells}+\mathrm{Act}.\;\mathrm{supernatant}}\times 100$$

#### 3.6.3. Binding Affinity Studies

Cells (monolayer, equivalent to 7 × 10^6^ cells per flask) were incubated for 1 h (37 °C, 5% CO_2_) with a fixed activity of each complex under study (0.925 MBq, 25 µCi) and with increasing concentrations of flutamide (25 µL, 1.2 × 10^−3^ M–1.2 × 10^−8^ M). After incubation, the culture medium was removed, and cells were washed with PBS and treated with trypsin (5 min, 37 °C). The activity in the supernatant and the activity bound to the cells were measured. Uptake values (expressed as percentages) were plotted against a log of the concentration of flutamide. The IC_50_ values were calculated with a nonlinear regression analysis with the GraphPad Prism 9 computer system (Boston, MA, USA).

## 4. Conclusions

The syntheses of four flutamide derivatives bearing different chelating units for coordinating ^99m^Tc through the formation of tricarbonyl complexes were successfully achieved with high purity and adequate yield. The technetium complexes were also obtained with high radiochemical purity after HPLC purification, and their structures were corroborated with mass spectroscopy using stable Re as a surrogate. **C1**, **C3**, and **C4** were highly stable in the labeling milieu, in human plasma, and against histidine as a competing ligand. The studies in AR-positive LNCaP cells and AR-negative PC3 cells indicated that the three complexes were taken up via interaction with the androgen receptor; however, the IC_50_ values were two orders of magnitude lower than those of flutamide. Moreover, **C3** was also taken up in a significant amount by PC3 cells, indicating that the specificity of this tracer for molecular imaging of the AR would not be enough. In spite of this result, the data derived from our study on the derivatization, labeling, and evaluation of biomolecules could be utilized for the designing of potential radiopharmaceuticals for this or other molecular targets.

## Figures and Tables

**Table 1:** Optimized preparation conditions of ^99m^Tc complexes.

Complex	Mass (mg)	Dissolution Solvent	Incubation Temp. (°C)	Incubation Time (min)	RCP (%)
**C1**	3	Methanol	75	30	80–85%
**C2**	5	Water/HCl	75	30	80–85%
**C3**	4	Acetonitrile	25	30	75–85%
**C4**	4	Acetonitrile	25	30	55–65%

**Table 2:** MS results of the rhenium analogs of **C1**–**C4**.

Complex	MW *	
**C1**-Re	672	MS (ESI, 10 eV): *m*/*z* (MW C1 = 672) {[C1 + H]^+^} 673
**C2**-Re	637	MS (ESI, 10 eV): *m*/*z* (MW C1 = 637) {[C2 + H]^+^} 638
**C3**-Re	716	MS (ESI, 10 eV): *m*/*z* (MW C3 = 716) {[C3 + H]^+^} 717
**C4**-Re	743	MS (ESI, 10 eV): *m*/*z* (MW C4 = 743) {[C4 + H]^+^} 744

* Molecular weight was calculated considering only ^187^Re as the principal isotope of stable rhenium. The results of the MS spectra matched the isotope distribution of ^186^/^187^Re in stable rhenium.

**Table 3:** Lipophilicity and plasma protein binding (PPB%) at 1 h incubation of ^99m^Tc complexes. Values are expressed as means ± SD, n = 3.

Complex	Log P	PPB (%) at 1 h
**C1**	1.2 ± 0.1	39.3 ± 0.8
**C2**	0.75 ± 0.04	49.0 ± 2.5
**C3**	1.5 ± 0.2	14.2 ± 0.9
**C4**	1.1 ± 0.1	45.1 ± 1.4

**Table 4:** Plasma protein binding percentage (PPB%) for complex **C2**. Values are expressed as means ± SD, n = 3.

Time (min)	PPB%
30	12.0 ± 0.6
60	49.0 ± 2.5
90	61.5 ± 30.1

**Table 5:** Uptake values in LNCaP cells evaluated at different times. Values are expressed as means ± SD, n = 3.

Time (h)	C1 (%)	C3 (%)	C4 (%)
1	23.9 ± 1.2	56.5 ± 1.8	27.8 ± 1.7
2	26.9 ± 1.3	54.8 ± 2.4	26.1 ± 1.8
4	25.3 ± 0.7	56.4 ± 3.1	25.7 ± 2.2

**Table 6:** Comparison of the uptakes of ^99m^Tc complexes at 1 h incubation in AR-positive cells (with and without blocking) and AR-negative cells. Values are expressed as means ± SD, n = 3.

Complex	Uptake in LNCaP Cells (%)	Uptake in Blocked LNCaP Cells (%)	Uptake in PC3 Cells
**C1**	23.9 ± 1.2	12.5 ± 3.1	14.5 ± 2.4
**C3**	56.5 ± 1.8	17.7 ± 4.2	41.4 ± 2.4
**C4**	27.8 ± 1.7	11.3 ± 2.8	16.9 ± 2.0

**Table 7:** Efflux (%) values of the ^99m^Tc complexes in LNCaP cells as a function of incubation time. Values are expressed as means ± SD, n = 3.

Time (h)	C1	C3	C4
1	34.2 ± 2.1	18.6 ± 1.6	47.9 ± 1.7
2	34.3 ± 2.4	19.9 ± 1.4	49.0 ± 1.5
4	33.9 ± 2.2	18.9 ± 1.2	48.4 ± 1.8

**Table 8:** IC_50_ values, expressed as means ± SD, n = 3.

Complex	IC_50_ (µM)
**C1**	55.8 ± 7.6
**C3**	36.8 ± 9.6
**C4**	41.2 ± 11.2

## Data Availability

Not applicable.
